# Signature miRNAs in colorectal cancers were revealed using a bias reduction small RNA deep sequencing protocol

**DOI:** 10.18632/oncotarget.6460

**Published:** 2015-12-04

**Authors:** Guihua Sun, Ya-Wen Cheng, Lily Lai, Tsui-Chin Huang, Jinhui Wang, Xiwei Wu, Yafan Wang, Yasheng Huang, Jinghan Wang, Keqiang Zhang, Shuya Hu, Ji-Rui Yang, Yun Yen

**Affiliations:** ^1^ Department of Molecular Pharmacology, Beckman Research Institute of the City of Hope, Duarte, CA, USA; ^2^ Department of Surgery, Beckman Research Institute of the City of Hope, Duarte, CA, USA; ^3^ Integrated Genome Core, Beckman Research Institute of the City of Hope, Duarte, CA, USA; ^4^ Graduate Institute of Cancer Biology and Drug Discovery, College of Medical Science and Technology, Taipei Medical University, Taipei, Taiwan

**Keywords:** microRNA, colorectal cancer, small RNA deep sequencing, miR-21, miR-143

## Abstract

To explore the role of miRNAs in colorectal cancers (CRC), we have deep sequenced 48 pairs of frozen CRC samples, of which 44 pairs produced high quality sequencing data. By using a combined approach of our bias reduction small RNA (smRNA) deep sequencing protocol and Illumina small RNA TruSeq method for sample bar coding, we have obtained data from samples of relatively large size with bias reduced digital profile results. This novel approach allowed us to validate many previously published results using various techniques to profile miRNAs in CRC tissues or cell lines and to characterize ‘true’ miRNA signatures highly expressed in colon/rectum (CR) or CRC tissues. According to our results, miR-21, a miRNA that is up-regulated in CRC, and miR-143, a miRNA that is down-regulated in CRC, are the two miRNAs that dominated the miRNA population in CR tissues, and probably are also the most important miRNAs in CRCs. These two miRNAs, together with the other eight miRNAs, miR-148a, -194, -192, 200b, -200c, -10b, -26a, and -145, with descending expressing levels, constituted the top 10 highly expressed miRNAs in CR/CRC. Using TaqMan miRNA qPCR, we detected the relative expression of some of the CRC miRNAs in 10 CRC cell lines, validated their dysregulation under cancer condition, and provided possible explanation for their dysregulation, which could be caused by APC, KRAS, or TP53 mutations. We believe these results will provide a new direction in future miRNA-related CRC development studies, and application of miRNAs in CRC diagnosis/prognosis, and therapy.

## INTRODUCTION

CRC includes two major entities: colon cancer (CC) and rectal cancer (RC). It ranks the 3^rd^ in all cancer cases worldwide and represents one of the cancers with high mortality [1]. CRC rates are much higher in populations consuming low fiber, high fat, and high protein diet, compared with populations consuming high fiber, low fat, and low protein diet [2]. It is conceivable that as more populations in the world are moving towards high standard of living, which allows consuming varieties of foods, more CRC cases will be inevitably increasing worldwide. One supporting evidence for the above conclusion is that CRC already becomes the number one cancer cases in some rich areas that allows consuming large amount of high protein, high fat foods. CRC can be cured if it is diagnosed early. But because of its location, it is not easily spotted early and currently is usually diagnosed through unpleasant colonoscopy and biopsy (most in advanced stages already). It is conceivable researches that address novel mechanisms in CRC development may be able to provide noninvasive, cost-effective approaches to diagnose CRC in early stages and provide new avenues for treatment.

MicroRNA (miRNA) is a family of conserved small RNAs (smRNAs) that can regulate target gene expression through mediating mRNA degradation or translational repression. It was proposed that miRNAs will become new frontier players in cancer biology [3]. Reduced accumulation of specific miRNAs in colorectal neoplasia was reported early on [4]. The milestone miRNA-cancer paper that reported the profile of miRNAs in cancers to be correlated with the origin, progression, and metastasis of many cancers have attracted the attention of more cancer biologists in this new field. Since then, more and more miRNA-cancer studies have advanced basic cancer research, the development of biomarkers for diagnosis/prognosis, and the identification of miRNAs as targets for cancer therapy [5].

There are thousands of human miRNAs and their abundance is varied among different tissues [6, 7]. It is more practical to use a panel of miRNAs that are specifically expressed in certain cancer to study their role in carcinogenesis and to use them as biomarkers in cancer clinical practice. Therefore, it is critical to profile miRNAs that are highly or specifically expressed in certain cancer. While both microarray and deep sequencing are excellent platforms for global miRNA profiling, they are labor intensive, costly, have relatively low detecting range when compared to RT-qPCR, and the turnaround time is in days. In clinical setting, a small number of highly expressed miRNAs in the panel that can be detected by qRT-PCR within a few hours will be ideal. So the ideal approach is to screen a panel of miRNA using global miRNA profiling approaches, such as deep sequencing and microarray, then detect this panel of miRNAs by qRT-PCR that has high specificity, sensitivity, and fast turnaround time.

To explore the role of miRNAs in CRCs, Northern blotting, miRNA microarray, miRNA qRT-PCR, and high-throughput small RNA sequencing methods have been applied in miRNA-CRC studies using CRC cell lines or patient samples in the past several years (See latest review [8]). Although results from these published studies are encouraging, they are limited by the techniques available, sample size, and the difficulty in sampling CRCs at different stages [8-10]. We are still in the early stage of collecting CRC-miRNA data and much more of this kind of data are necessary to advance this field and achieve our goal of using miRNAs in CRC diagnosis/prognosis, and therapy. To achieve this goal, CR/CRC miRNAs profiling will be the first step. Among all the miRNA profiling platforms, deep sequencing is the best technique to identify signature/marker miRNAs because they can detect thousands of annotated and novel miRNAs, as well as other small RNAs simultaneously.

Here we report our finding of a pilot study on miRNA deep sequencing using 48 paired frozen biopsy CRC samples, of which 44 paired samples produced high sequencing quality data and were used for further analysis. This study allowed us to validate many previously published results. Moreover, by using a bias reduction protocol for smRNA deep sequencing, we were able to provide novel digital miRNA profile data of CRC samples. The bias reduced digital profile results allowed us to characterize a ‘true’ miRNA signature that is highly expressed in CR or CRC tissues. Using TaqMan miRNA qPCR, we detected the relative expression of some of the CRC miRNAs obtained from deep sequencing in 10 CRC cell lines, which validated their dysregulation under cancer condition. We also provide possible explanation for their dysregulation, which could be caused by APC, KRAS, or TP53 mutations. We believe these results will provide a new direction in future miRNA-CRC development studies, and the application of miRNAs in CRC diagnosis/prognosis, and therapy.

## RESULTS

### Deep sequencing data summary and signature miRNAs in CR or CRC

Today, the bias results in ligation-based deep sequencing gene profiling remains as one major technical problem for its application to quantity gene expression after its discovery and publication of our bias reduction workaround solution [11-13]. In the current study, CRC samples were sequenced using a combined approach of our bias reduction smRNA deep sequencing method with the protocol provided in the Illumina TRUE smRNA sequencing kit for both bias reduction and sample bar coding [11]. Total 48 paired samples were used for this pilot experiment (one and half runs, each run using twelve lanes on an Illumina HighSeq2000 machine with eight bar coded samples per lane). After data validation, we were able to get 46 paired samples (46 samples set) that produced high quality sequencing data.

**Figure 1:**
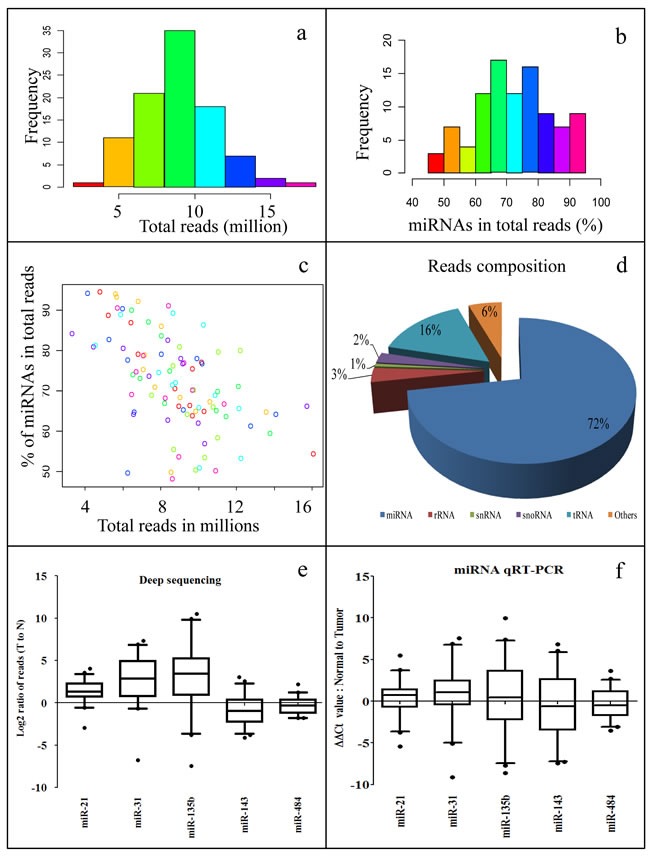
Summary of smRNA deep sequencing reads **a**. Histogram of smRNA deep sequencing reads for all samples. Majority of samples have smRNA reads between 7 to 10 millions. **b**. Histogram of miRNA percentage for all samples. Most of the samples have miRNA percentage ranging from 60% to 80%. **c**. Paired x-y plot of total smRNA reads per samples versus % of miRNA population per sample. **d**. Summary of smRNA composition for all samples. **e**. Box plot for log transformed T to N ratio of deep sequencing reads of miR-21, -31, -135b, -143, and -484 in the 44 pairs sample set. **f**. Box plot for ΔΔCt value of miRNA qRT-PCR detected miR-21, -31, -135b, -143, and -484 in the 44 pairs sample set.

After filtering out low quality reads, we obtained about 7 to 10 M smRNA reads per sample for most of the samples (Figures 1a, S1, Table S1). Analysis of reads composition of smRNA fragments revealed that majority of them are mature miRNAs, ranging from 60 to 80 % for most of the samples (Figures 1b, S2). Total reads agreed well with mature miRNA reads in each sample (Figure 1c). Summarized composition of smRNAs in reads from all sequenced samples showed that miRNAs represent the largest group, comprising of 72% of the total population. The second largest population is tRNA-derived smRNAs, which consist of 16% of the total population (Figure 1d). This data showed that we have high quality RNA samples and small RNA libraries, and our smRNA deep sequencing produced highly reliable data. Next, we performed miRNA qRT-PCR for five miRNAs to validate our smRNA deep sequencing results. The qRT-PCR data agrees well with the deep sequencing reads (Figure 1e, 1f). Two pairs of samples were later reclassified as none cancer samples by pathologist and were removed from further analysis (44 samples set in Table 1; most data analysis refers to this set hereafter unless specified).

**Table 1:** Summary information of CRC samples used in this study

Samples		All	Colon	Rectal
Size	Normal	44	29	15
	Tumor	44	29	15
Stage	0	1	1	0
	1	7	5	2
	2	7	5	2
	3	33	22	11
	4	1	1	0
Sex	Male	13	7	6
	Female	31	23	8
Race	Caucasian	32	19	13
	African	4	3	1
	Asian	7	6	1
	Unknown	1	1	0
Age	30-39	2	1	1
	40-49	2	1	1
	50-59	16	10	6
	60-69	9	7	2
	70-79	10	6	4
	80-89	5	4	1

**Figure 2:**
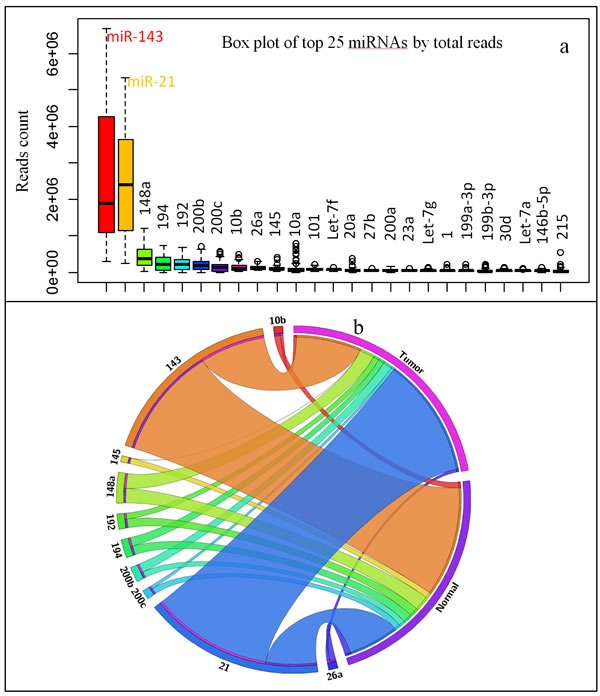
Highly expressed miRNAs in CR/CRC **a**. Box plot of top 25 miRNAs by total reads in 44 pairs sample set. **b**. Circular plot of top 10 miRNA in normal tissue versus tumor tissue (44 pairs sample set).

Our bias reduction approach showed miR-21 and miR-143 together represent over 60% of all miRNAs, and the rest of the eight miRNAs from the top 10 highly expressed miRNAs covers 17% of total miRNAs (Figure 2a). These data implied CR tissues are mainly controlled by miR-21 and miR-143. While miR-21 is up-regulated, miR-143 is down-regulated in majority of the tumor samples, which results as the rank of their abundance was switched in tumor samples versus normal samples (Figure 2b). Only the rank of miR-148a remains unchanged (by %, Tumor versus Normal) in the top 10 miRNAs that are highly expressed in CR/CRC (Tables 2, S2).

**Table 2:** Top 25 miRNAs in samples by reads or percentage

	By Reads ( in 1000, Sorted by All)					By % in all reads (Sorted by All)			
Rank	miRNA	Normal	Tumor	All	*p*-value	miRNA	Normal	Tumor	All
1	143	1831.29	1058.94	1445.11	0.0028	143	38.13	21.72	29.86
2	21	817.00	1993.57	1405.28	0.0000	21	17.01	40.89	29.04
3	148a	232.12	233.52	232.82	0.8591	148a	4.83	4.79	4.81
4	194	165.30	103.07	134.18	0.0048	194	3.44	2.11	2.77
5	192	146.86	96.18	121.52	0.0075	192	3.06	1.97	2.51
6	200b	110.35	109.81	110.08	0.6898	200b	2.30	2.25	2.27
7	200c	80.28	72.69	76.49	0.4658	10b	1.93	1.00	1.46
8	10b	92.64	48.62	70.63	0.0000	26a	1.88	1.04	1.46
9	26a	90.08	50.85	70.46	0.0000	145	1.70	0.67	1.18
10	145	81.46	32.82	57.14	0.0495	200c	1.67	1.49	1.58
11	10a	55.01	55.68	55.34	0.8860	10a	1.15	1.14	1.14
12	101	53.57	41.33	47.45	0.0038	101	1.12	0.85	0.98
13	let-7f	38.86	35.08	36.97	0.0774	let-7f	0.81	0.72	0.76
14	20a	23.45	46.18	34.82	0.0032	1	0.76	0.32	0.54
15	27b	34.50	26.95	30.73	0.0002	27b	0.72	0.55	0.64
16	200a	30.00	29.18	29.59	0.6801	let-7g	0.67	0.49	0.58
17	23a	27.68	28.95	28.31	0.6455	215	0.66	0.20	0.43
18	let-7g	32.19	23.93	28.06	0.0001	378	0.64	0.25	0.44
19	199a-3p	24.32	29.85	27.09	0.1416	200a	0.62	0.60	0.61
20	199b-3p	24.32	29.85	27.09	0.1416	30d	0.59	0.41	0.50
21	1	36.33	15.68	26.00	0.0001	23a	0.58	0.59	0.59
22	30d	28.36	20.12	24.24	0.0023	let-7a	0.54	0.46	0.50
23	let-7a	25.71	22.37	24.04	0.0439	199a-3p	0.51	0.61	0.56
24	146b-5p	20.28	27.00	23.64	0.1105	199b-3p	0.51	0.61	0.56
25	378	30.54	12.25	21.40	0.0000	20a	0.49	0.95	0.72

### Compare published profile data with results in the current study

The advantage for miRNA deep sequencing is that the status (up/down in expression level) of miRNA dysregulation can be directly linked with the expression level of miRNAs in all CR/CRC miRNAs and the result can be digitalized. We compared our profile data with published CRC-miRNA results (Data was summarized in reference [8-10]). These top 10 miRNAs (by %) we identified in this study are also among the highly expressed miRNAs in CR/CRC reported before albeit in a different rank [14-16]. Overall, the status of miRNA dysregulation in published data agreed well with the results in our study (Tables 3, S3). However, several of dysregulated miRNAs that were reported previously are expressed at very low level according to our data, with only few reads per sample on average. This comparison showed the limitation of previous profiling methods. Therefore, some of the previously reported miRNAs may not play a critical physiological role in CR/CRC and will be difficult for detection and clinical applications due to their low abundance (Table 3). Furthermore, many of the reported dysregulated miRNAs are up/down less than 50%, implying that they will not be good candidates to be used as biomarkers (Table S3).

**Table 3:** Dysregulated CRC miRNAs: published list versus this study (changes by > 50%)

Rank	miRNA	Normal	Tumor	All (T+N)	Ref	This	T/N ratio
161	135b	94	733	414	Up	Up	7.80
596	663	1	4	3	Down	Up	4.00
149	224	239	815	527	Up	Up	3.41
101	183	667	2174	1421	Up	Up	3.26
79	182	1641	5197	3419	Up	Up	3.17
2	21	817001	1993567	1405284	Up	Up	2.44
228	96	76	168	122	Up	Up	2.21
34	17	10882	23553	17218	Up	Up	2.16
39	7	8042	17185	12614	Up	Up	2.14
14	20a	23452	46183	34818	Up	Up	1.97
53	203	6086	10185	8136	Up	Up	1.67
93	106a	1513	2322	1918	Up	Up	1.53
5	192	146863	96181	121522	Down	Down	0.65
73	99b	5486	3549	4518	Down	Down	0.65
37	125a-5p	17052	10790	13921	Down	Down	0.63
4	194	165296	103069	134183	Down	Down	0.62
52	30c	11117	6625	8871	Down	Down	0.60
84	195	3654	2123	2889	Down	Down	0.58
1	143	1831286	1058943	1445115	Down	Down	0.58
80	133a	4218	2340	3279	Down	Down	0.55
42	451	15459	8048	11754	Down	Down	0.52
115	29c	1449	754	1102	Down	Down	0.52
43	30a	15825	7575	11700	Down	Down	0.48
63	375	7856	3399	5628	Down	Down	0.43
21	1	36329	15680	26005	Down	Down	0.43
423	129-3p	17	7	12	Down	Down	0.41
10	145	81457	32816	57137	Down	Down	0.40
25	378	30542	12252	21397	Down	Down	0.40
411	139-3p	20	8	14	Down	Down	0.40
147	9	765	294	530	Down	Down	0.38
318	135a	60	21	41	Up	Down	0.35
26	215	31876	9542	20709	Down	Down	0.30
410	124	23	5	14	Down	Down	0.22
378	137	31	6	19	Down	Down	0.19

### The power of paired samples and miRNAs pool to distinguish tumor tissues from normal tissues

**Figure 3:**
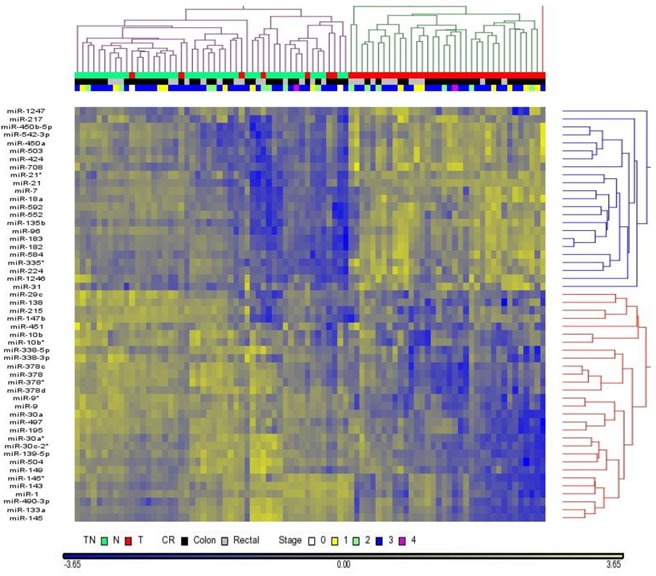
Hierarchical cluster analysis classifies tumor versus normal tissues Dendrogram of miRNAs with p<0.05 cluster normal tissue versus tumor tissue (44 pairs sample set). miRNAs are clustered into two groups that are either correlated with normal tissue or tumor tissue. There are several cases of tumor tissues are classified into normal tissues.

There are hundreds of miRNAs detected in our samples and the variation of reads is large, it is unlikely all of them can be used to classify tumor versus normal tissues. Unsupervised hierarchical cluster analysis using reads count of all miRNAs to distinguish tumor versus normal tissues failed to give a clear results. Instead, normal tissues were mainly clustered into two groups (Figure S3). To reduce the variation among samples, we applied the power of paired sample by using tumor to normal reads count ratio (TN ratio) in each paired sample and selected a list of miRNAs using the criteria of p-value < 0.05. Using this approach, we were able to differentiate most tumor samples from normal samples and these differentially expressed miRNAs are classified into two groups that are up-regulated or down-regulated in tumor samples, compared with normal samples (Figure 3).

### Detect the CRC miRNAs in CRC cell lines

**Figure 4:**
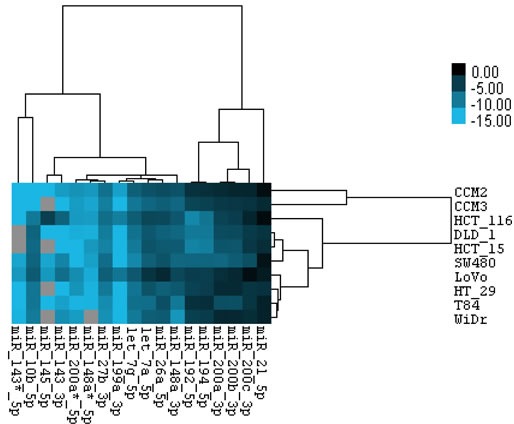
Relative expression level of miRNAs in CRC cell lines Total of 18 miRNAs expression were measured by Taqman miRNA qPCR. ΔCt value of each miRNA was calculated as average value of ΔCt_miRNA_ = Ct_cell line-miRNA_ − Ct_cell line-U6sn_ from three independent reactions with duplicates in each reaction, and −ΔCt_miRNA_ values were used for hierarchical clustering.

Cancer cell lines have advantages over tissues for many cancer-related studies and research, such as drug testing, to study cancer causes by gene mutations, and using tumor mouse model for cancer studies. Therefore, we detected some CRC miRNAs from our deep sequencing results in 10 CRC cell lines using miRNA Taqman qPCR and correlated their expression status with APC, KRAS and TP53 mutations. Expression levels were measured by relative –delta Ct value (miRNA to U6sn). The expression level of miR-21, miR-200 family, miR-194, miR -192, miR-148a, and miR-26a, was relatively high, consistent with their high expression in CRC tissues; the expression level of miR-143-3p, miR-10b, and miR-145 was relative low, agreeing with their down-regulation in CRC tissues. Heatmap analysis revealed that mutation in either TP53 (HCT116 and LoVo are wild type) or APC (HCT116 is wild type) may be responsible for the down-regulated miRNAs (Figures 4, S4, S5). These miRNAs may also be used to separate k-ras mutation from wt (HT-29 and WiDr are wild type) (Figures 4, S6). These data provide the opportunity to manipulate these miRNAs in CRC cell lines for loss or gain of miRNA function studies using cell lines and tumor mouse model.

### Functional enrichment analysis of miR-21, miR-200c, and miR-26a target genes and pathways

To explore the possible role of oncogenic miRNAs in CRC, we performed enrichment analysis of experimentally validated target genes of miRNAs in oncogenic signatures and biological pathways listed in MSigDB. We listed the top 5 enriched terms in each databases in Table 4. The results demonstrated that miR-21-regulated genes are involved in RAS/RAF activation, including mitogen-activated signaling and TBK1-regulated pathways, miR-200 family-targeted genes are suppressed by VEGF signaling, and miR-26a-modulated genes are correlated with cell cycle progression.

**Table 4:** Top 5 gene sets in oncogenic signature, Reactome, and Biocarta overlapped with experimental validated miR target genes

miR	Gene Set Name	# Genes in Gene Set (K)	# Genes in Overlap (k)	k/K	*p*-value^a^	FDR q-value^b^
miR-21	TBK1.DF_DN	287	32	0.1115	2.41E-23	4.56E-21
	P53_DN.V1_UP	194	18	0.0928	2.51E-12	1.58E-10
	RAF_UP.V1_DN	194	18	0.0928	2.51E-12	1.58E-10
	TBK1.DF_UP	290	19	0.0655	2.66E-10	1.26E-08
	PIGF_UP.V1_UP	191	15	0.0785	1.74E-09	6.23E-08
	REACTOME_METABOLISM_OF_LIPIDS_AND_LIPOPROTEINS	478	22	0.046	7.82E-09	2.72E-06
	REACTOME_DEVELOPMENTAL_BIOLOGY	396	20	0.0505	8.06E-09	2.72E-06
	REACTOME_FATTY_ACID_TRIACYLGLYCEROL_AND_KETONE_BODY_METABOLISM	168	12	0.0714	2.09E-07	4.69E-05
	REACTOME_SIGNALING_BY_TGF_BETA_RECEPTOR_COMPLEX	63	8	0.127	2.85E-07	4.80E-05
	REACTOME_HEMOSTASIS	466	19	0.0408	5.23E-07	5.92E-05
	BIOCARTA_MAPK_PATHWAY	87	13	0.1494	6.50E-12	1.41E-09
	BIOCARTA_HIVNEF_PATHWAY	58	10	0.1724	4.16E-10	4.51E-08
	BIOCARTA_KERATINOCYTE_PATHWAY	46	9	0.1957	9.62E-10	6.96E-08
	BIOCARTA_CTCF_PATHWAY	23	7	0.3043	2.53E-09	1.37E-07
	BIOCARTA_ALK_PATHWAY	37	8	0.2162	3.59E-09	1.56E-07
miR-200c	TBK1.DF_DN	287	4	0.0139	2.53E-04	2.71E-02
	BMI1_DN_MEL18_DN.V1_UP	145	3	0.0207	5.10E-04	2.71E-02
	ESC_V6.5_UP_LATE.V1_DN	186	3	0.0161	1.05E-03	2.71E-02
	P53_DN.V1_DN	192	3	0.0156	1.15E-03	2.71E-02
	VEGF_A_UP.V1_DN	193	3	0.0155	1.17E-03	2.71E-02
	REACTOME_INTRINSIC_PATHWAY_FOR_APOPTOSIS	30	3	0.1	4.53E-06	3.05E-03
	REACTOME_HEMOSTASIS	466	6	0.0129	1.02E-05	3.43E-03
	REACTOME_VEGF_LIGAND_RECEPTOR_INTERACTIONS	10	2	0.2	4.98E-05	9.68E-03
	REACTOME_RIG_I_MDA5_MEDIATED_INDUCTION_OF_IFN_ALPHA_BETA_PATHWAYS	73	3	0.0411	6.72E-05	9.68E-03
	REACTOME_PLATELET_ACTIVATION_SIGNALING_AND_AGGREGATION	208	4	0.0192	7.36E-05	9.68E-03
	BIOCARTA_VEGF_PATHWAY	29	3	0.1034	4.08E-06	8.86E-04
	BIOCARTA_AKAP13_PATHWAY	12	2	0.1667	7.30E-05	5.97E-03
	BIOCARTA_HIF_PATHWAY	15	2	0.1333	1.16E-04	5.97E-03
	BIOCARTA_RELA_PATHWAY	16	2	0.125	1.32E-04	5.97E-03
	BIOCARTA_IL7_PATHWAY	17	2	0.1176	1.50E-04	5.97E-03
miR-26a	RB_DN.V1_UP	137	8	0.0584	6.20E-08	1.17E-05
	E2F1_UP.V1_UP	189	7	0.037	8.79E-06	5.73E-04
	CYCLIN_D1_KE_.V1_UP	190	7	0.0368	9.09E-06	5.73E-04
	CAMP_UP.V1_UP	200	7	0.035	1.27E-05	5.99E-04
	MTOR_UP.N4.V1_UP	196	6	0.0306	1.12E-04	4.24E-03
	REACTOME_CELL_CYCLE	421	18	0.0428	5.79E-14	3.90E-11
	REACTOME_CELL_CYCLE_MITOTIC	325	14	0.0431	3.62E-11	1.22E-08
	REACTOME_MITOTIC_G1_G1_S_PHASES	137	10	0.073	1.50E-10	3.36E-08
	REACTOME_G1_S_TRANSITION	112	8	0.0714	1.28E-08	2.15E-06
	REACTOME_CYCLIN_E_ASSOCIATED_EVENTS_DURING_G1_S_TRANSITION	65	6	0.0923	1.92E-07	2.48E-05
	BIOCARTA_G1_PATHWAY	28	5	0.1786	6.92E-08	1.50E-05
	BIOCARTA_CELLCYCLE_PATHWAY	23	4	0.1739	1.70E-06	1.23E-04
	BIOCARTA_CTCF_PATHWAY	23	4	0.1739	1.70E-06	1.23E-04
	BIOCARTA_WNT_PATHWAY	26	4	0.1538	2.84E-06	1.54E-04
	BIOCARTA_ALK_PATHWAY	37	4	0.1081	1.22E-05	5.28E-04

a P-value from the hypergeometric distribution for (k-1, K, N - K, n) where k is the number of genes in the intersection of the query set with a set from MSigDB, K is the number of genes in the set from MSigDB, N is the total number of all known human gene symbols, and n is the number of genes in the query set.

b False discovery rate analog of hypergeometric p-value after correction for multiple hypothesis testing according to Benjamini and Hochberg.

**Figure 5:**
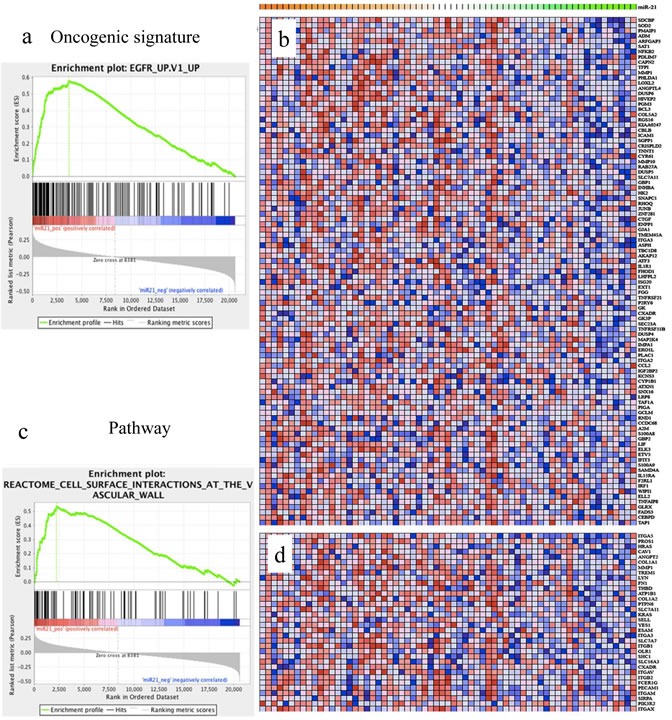
miR-21 expression is positive correlated with cancer-associated pathways Top ranked enriched terms of gene set enrichment analysis (GSEA) on miR-21 correlated gene expression. **a**. Oncogenic signature (NES = 1.935, FDR = 0.061). **b**. The expression levels of leading edge genes in oncogenic signature and miR-21 with descending levels from red to blue and orange to green, respectively. **c**. Reactome pathway database (NES = 1.921, FDR = 0.247). **d**. The expression levels of leading edge genes in reactome pathway database and miR-21 with descending levels from red to blue and orange to green, respectively.

Furthermore, we analyzed the co-expressed genes and miRNAs in CRC patients from an independent dataset in terms of oncogenic signature and signaling transduction pathway using gene set enrichment analysis (GSEA, Table 5). Of interest, our results revealed that genes involving EGFR, TBK1, KRAS signaling, as well as cell surface interactions at the vascular wall were positively correlated with miR-21 expression (Figure 5), supporting its oncogenic roles with integrated oncogenic gene expression in CRC progression.

**Table 5:** Top 3 enriched terms of miR correlated gene expression in oncogenic signatures and signaling pathway database Reactome

miR	Name	Size	ES	NES	*p*-val	FDR q-val
miR-21	Oncogenic signature					
	EGFR_UP.V1_UP*	177	0.578	1.935	0.002	0.061
	TBK1.DN.48HRS_DN*	49	0.519	1.666	0.024	0.210
	KRAS.DF.V1_UP*	174	0.444	1.663	0.029	0.180
	YAP1_DN	40	−0.480	−1.741	0.006	0.448
	KRAS.600_UP.V1_DN	266	−0.379	−1.469	0.048	1.000
	KRAS.AMP.LUNG_UP.V1_UP	128	−0.418	−1.462	0.066	1.000
	Pathway					
	REACTOME_CELL_SURFACE_INTERACTIONS_AT_THE_VASCULAR_WALL*	84	0.534	1.921	0.002	0.247
	REACTOME_IL1_SIGNALING	32	0.628	1.864	0.002	0.275
	REACTOME_SIGNALING_BY_ILS	98	0.505	1.748	0.015	0.785
	REACTOME_DEFENSINS*	26	−0.636	−1.913	0.004	0.242
	REACTOME_ACYL_CHAIN_REMODELLING_OF_PC	16	−0.604	−1.769	0.011	0.690
	REACTOME_ACYL_CHAIN_REMODELLING_OF_PE	17	−0.594	−1.768	0.010	0.467
miR-200c	Oncogenic signature					
	JAK2_DN.V1_DN	132	0.410	1.518	0.079	1.000
	SNF5_DN.V1_DN	146	0.332	1.408	0.057	1.000
	CAHOY_OLIGODENDROCUTIC	89	0.291	1.216	0.176	1.000
	RPS14_DN.V1_UP	179	−0.528	−1.619	0.067	1.000
	SNF5_DN.V1_UP	163	−0.440	−1.612	0.048	1.000
	KRAS.LUNG.BREAST_UP.V1_UP	134	−0.389	−1.420	0.092	1.000
	Pathway					
	REACTOME_CHOLESTEROL_BIOSYNTHESIS	21	0.688	1.545	0.076	1.000
	REACTOME_TRANSPORT_OF_VITAMINS_NUCLEOSIDES_AND_RELATED_MOLECULES	31	0.410	1.454	0.030	1.000
	REACTOME_BIOLOGICAL_OXIDATIONS	120	0.323	1.314	0.119	1.000
	REACTOME_GPVI_MEDIATED_ACTIVATION_CASCADE	30	−0.593	−1.807	0.004	1.000
	REACTOME_GENERATION_OF_SECOND_MESSENGER_MOLECULES	24	−0.754	−1.801	0.014	0.780
	REACTOME_COSTIMULATION_BY_THE_CD28_FAMILY	57	−0.552	−1.791	0.012	0.576
miR-26a	Oncogenic signature					
	TBK1.DN.48HRS_DN*	49	0.602	1.920	0.000	0.056
	BCAT_BILD_ET_AL_UP	44	0.465	1.520	0.046	1.000
	BCAT_GDS748_DN	40	0.411	1.511	0.020	1.000
	KRAS.AMP.LUNG_UP.V1_UP	128	−0.434	−1.536	0.035	1.000
	KRAS.LUNG.BREAST_UP.V1_UP	134	−0.394	−1.526	0.055	1.000
	KRAS.600.LUNG.BREAST_UP.V1_UP	260	−0.376	−1.510	0.048	0.809
	Pathway					
	REACTOME_TRANSCRIPTIONAL_REGULATION_OF_WHITE_ADIPOCYTE_DIFFERENTIATION	53	0.478	1.750	0.006	1.000
	REACTOME_ARMS_MEDIATED_ACTIVATION	15	0.650	1.688	0.006	1.000
	REACTOME_PROLONGED_ERK_ACTIVATION_EVENTS	17	0.610	1.660	0.013	1.000
	REACTOME_OLFACTORY_SIGNALING_PATHWAY	78	−0.636	−1.816	0.012	0.507
	REACTOME_LIGAND_GATED_ION_CHANNEL_TRANSPORT	21	−0.661	−1.691	0.022	0.979
	REACTOME_DEFENSINS	26	−0.575	−1.647	0.018	0.946

* Bold indicates significantly enriched terms with FDR less than 0.25.

## DISCUSSION

The role of miRNAs in CRC has attracted attention from researchers as well from physicians. MiRNAs in CRC tissues may correlate with it disease progression and disease stages and serve as biomarkers for diagnosis, prognosis, or suitable targets for treatments. Because miRNAs can also be transported through exosome and circulate exosomally, miRNAs have become very attractive noninvasive diagnosis biomarkers for cancers [17, 18]. In the past 10 years, many data have been generated using a variety of techniques and CRC samples [8-10]. Among all the techniques, deep sequencing is the most advanced and cost effective method to deal thousands of miRNAs at the same time, the ability to detect samples in a high dynamic range, and the potential to provide digital signature of miRNAs in profiled samples. It can also simultaneously detect novel miRNAs, others smRNAs, such as tRNA-derived smRNA fragments. But, due to the technical and bioinformatics challenges, and the high cost per run, only a few publications have employed deep sequencing technology in CRC-miRNA studies [14, 16].

In the current report, we performed a pilot study using deep sequencing technology to profile smRNAs from paired frozen CRC tissue samples. We also applied sample bar-coding to reduce cost and increase efficiency. Compared with published results, we get more reads (about 5 fold) and may have produced a list of ‘true’ signature miRNAs in CR/CRC. These signature miRNAs are critical candidate miRNAs that can be used in future miRNAs-CRC studies, CRC diagnosis/prognosis, and therapy. Their high expression level supports the notion that they could be biologically significant targets. The high level expression of these miRNAs may also reduce the technique challenge for detecting them to facilitate their application in clinical setting.

According to our results, miR-21, a miRNA that is up-regulated in CRC, and miR-143, a miRNA that is down-regulated in CRC, are the two miRNAs that dominated the miRNA population in CR tissue, and probably are also the most important miRNAs in CRC. The next eight miRNAs, from high to low in relative expression level, are miR-148a, -194, -192, 200b, -200c, -10b, -26a, and -145, which constituted the top 10 highly expressed miRNAs in CR/CRC. According to the changes in expression level, it seems miR-21 could act as an oncogene and miR-143 could serve a tumor suppressor role, but their exactly roles need to be defined by identifying their *bona fide* targets that can play direct physiology roles in CRC, and the cause of their dysregulation. The function of miR-21 has been well established because it is up-regulated in nearly all types of cancers and target tumor suppressor genes PTEN and PDCD4 [19-21]. The role of miR-143 in CRC is complicated despite many earlier reports indicating that miR-143 is a critical CRC miRNA [22-28], a recent careful study put this into question [29]. This previous study demonstrated that miR-143 and miR-145 are highly expressed in mesenchymal cells and are undetectable in colonic epithelial cells by various techniques, including the same deep sequencing platform we have used. Therefore, the role of miR-143 in CRC may be questionable. This result reminds us of a limitation of our study: the heterogeneity of tissue samples used in our study, especially the adjacent normal tissues that were resected during surgery. This may also have reflected in our clustering data (Figure S3). The current study can be further refined by detecting the list of identified candidate miRNAs by miRNA qRT-PCR using RNA samples from FFPE samples.

It also needs to be noted that some miRNAs with relatively low expression level, such as miR-31, miR-9, miR-135b, have been shown to play critical roles in CRC by several previous reports [22, 27, 30-35]. It is possible that these miRNAs may not need high expression level to be physiologically relevant. Alternatively, it could be technical difficulties that have prevented the detection of these miRNAs by ligation-based deep sequencing technology. One example is miR-31, which was detected as a low abundant miRNA using deep sequencing but can be detected relatively easy by qRT-PCR in this study and another independent study [16].

CRCs origins in the lining of the bowel and can invade the muscle layers underneath, and then grow through the bowel wall, and some will eventually develop into cancer. Our results showed that some muscle-specific-miRNAs (miR-1, miR-133a, ranked #21 and 80 respectively, Table S2) were detected to be highly expressed in CRC and could be involved in the progress of CRCs.

Another limitation of this study is that most of our samples are at stages 3, therefore it is impossible to draw a conclusion on how their expressions are related to CRC progression. However, by comparing the normal tissues to CRC tissues, our data support the conclusion that dysregulation of miRNA expression could contribute to diseases development. More stage 1, 2, and 4 samples will help to correlate miRNA expression to CRC development process and it can provide physicians treatment options in addition to tradition method.

Our Taqman miRNA qPCR expression data in CRC cell lines validated the some CRC miRNAs identified by deep sequencing. It will provide a basis for functional studies of miRNAs in CRC in the future.

## MATERIALS AND METHODS

### Patient cohort and samples collection

Paired samples (CRC and adjunct normal tissues) were collected during surgery at City of Hope using IRB protocol #COH05130. The biopsies were immediate frozen in liquid nitrogen after surgery and stored at −80°C until RNA isolation. Total 48 pairs of samples (CRC and adjunct region normal tissues) were used (Table 1). CRCs were staged according to American Joint Committee on Cancer (AJCC) staging criteria. Samples are most at stage 3, with age from 50 to 80, and ratio of colon to rectal about 2:1, and female to male ratio about 3:1.

### RNA isolation

Total RNA was isolated from frozen tissues or cell lines using Trizol (Life Technologies, Carlsbad, CA). RNA quality was checked and was quantified using a Nanodrop and an Agilent Bioanalyzer.

### Small RNA deep sequencing

One μg of total RNA was used to construct small RNA deep sequencing libraries as described in our previous publication [11] with the following modifications. Briefly, we mixed 64 equal molar oligos (adding three nt to the 3′ end of Illumina default 5′ adaptor) to produce a mixture of small RNA library 5′ ligation adaptor. We chose eight 3′ adaptors from a panel of 3′ adaptor in smRNA TruSeq kit (Illumina, San Diego, CA) as 3′ ligation adaptor and also bar-coding samples. Each lane was loaded with eight bar-coded samples on a HiSeq2000 (Illumina, San Diego, CA) machine using all eight lanes for the first run (64 samples) and four lanes for the second run (32 samples).

### Deep sequencing data analysis

Deep sequencing data analysis was performed as previously reported [11] with the following modification: 1) Reads were aligned to human genome hg19; 2) The mapping table was created using the human miRNA mature sequences from miRBase release16 and aligned back to human hg19 genome afterward [11]; 3) MiRNAs with less than 10 reads per sample in both normal and tumor samples are removed for further data analyzed. Deep sequencing data were analyzed, summarized, and plotted using R or Excel. Partek genomic suite (Partek Incorporated, St. Louis, Missouri) was used for hierarchical cluster analysis.

### SmRNA qRT-PCR for tissue samples

We followed the S-Poly(T) smRNA qRT-PCR detection protocol as previously reported [36]. Briefly, 100 ng total RNA was poly-A tailed using the poly-A tailing kit from Epicentre (Madison, WI). U47 snoRNA was chosen as RNA sample control. ΔΔCt value of each paired samples was calculated as (Ct_Tumor-miRNA_-Ct_Tumor-U47_) – (Ct_Normal-miRNA_-Ct_Normal-U47_).

### Taqman miRNA qPCR for cell lines

Taqman miRNA assay kits were purchased from Life Technologies (Grand Island, New York, USA). We followed the protocol from the manufacture. U6 snRNA was used as RNA sample control. ΔCt_miRNA_ values were used as their relative expression to U6 snRNA for data analysis.

### Functional gene set enrichment analysis

For miRNA-targeted genes, we obtained experimentally validated miRNA-target gene pairs (474, 49, and 175 experimentally validated target genes of miR-21-5p, miR-200c-3p, and miR-26a-5p, respectively) from miRTarBase [37] and assigned them as the query set for oncogenic signatures (C6), Reactome (CP:REACTOME of C2), and Biocarta (CP:BIOCARTA of C2) in MSigDB [38]. There are 189 and 674 gene sets collection in the oncogenic, and the Reactome category, respectively. For overlaps analysis, we used hypergeometric test to estimate the probability that the number of miR targeted genes overlapped with the genes from a given collection from the number of input genes randomly selected genes in 45956 human genes. For instance, the top term of miR-21 in oncogenic signature results in a probability of 4.56E-21 to draw 32 TBK1.DF_DN-associated genes or more from 474 randomly selected genes in the list. Significance is indicated by p-value from the hypergeometric test and adjusted by false discovery rate (FDR) after correction for multiple hypothesis testing according to Benjamini and Hochberg. For gene set enrichment analysis (GSEA), gene expression dataset was downloaded from GEO (GSE29623 [39]) with corresponding miRNA expression profiles. We permutated phenotype labels 1000 times and performed GSEA on the permutated data to obtain a random ES distribution. For the GSEA test, a p-value is calculated on the original data, and the resulting enrichment score is compared to the distribution of the values obtained from the permuted data. Distinct GSEA on miR-21, miR-26a, and miR-200c expression levels were performed on gene set in oncogenic signatures and Reactome using Pearson matrix. FDR < 0.25 was used to define significant enriched gene sets as suggested by GSEA documentation [40].

## SUPPLEMENTARY MATERIAL FIGURES


