# Supplementary material for: Signature miRNAs in colorectal cancers were revealed using a bias reduction small RNA deep sequencing protocol
Source: Oncotarget. 2015 Dec 4;7(4):3857–72. doi: 10.18632/oncotarget.6460 (PMC4826175; doi:10.18632/oncotarget.6460)
Supplement: Supplementary file 1 [file oncotarget-07-3857-s001.pdf]

Signature miRNAs in colorectal cancers were revealed using a bias reduction small RNA deep sequencing protocol

Supplementary Material

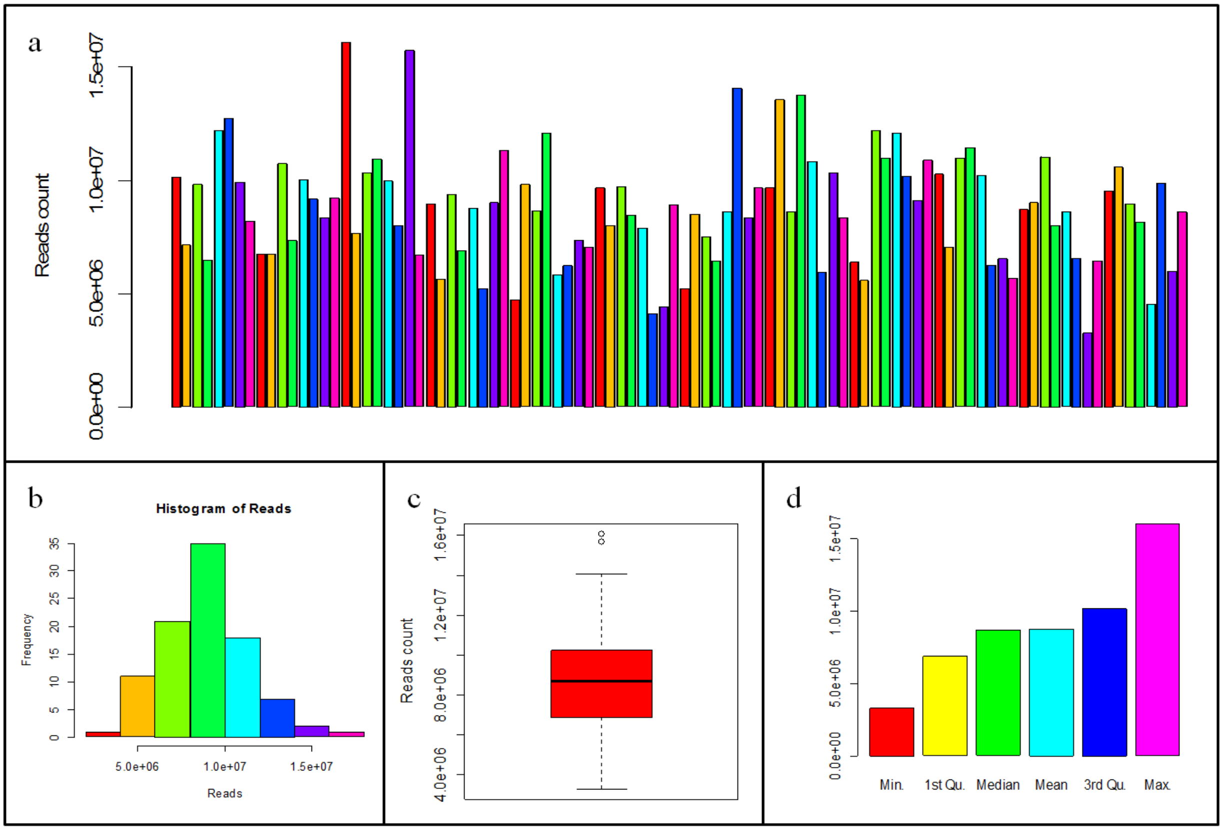

Figure S1. Total smRNA reads summary plots

a. Total smRNA reads count was plotted in each sample; b. Histogram graphs showing the distribution of total smRNA reads count in all samples; c. Box plot of total smRNA reads count in all samples; d. Box plot of the distribution of total smRNA reads count in all samples using the five-number summary method.

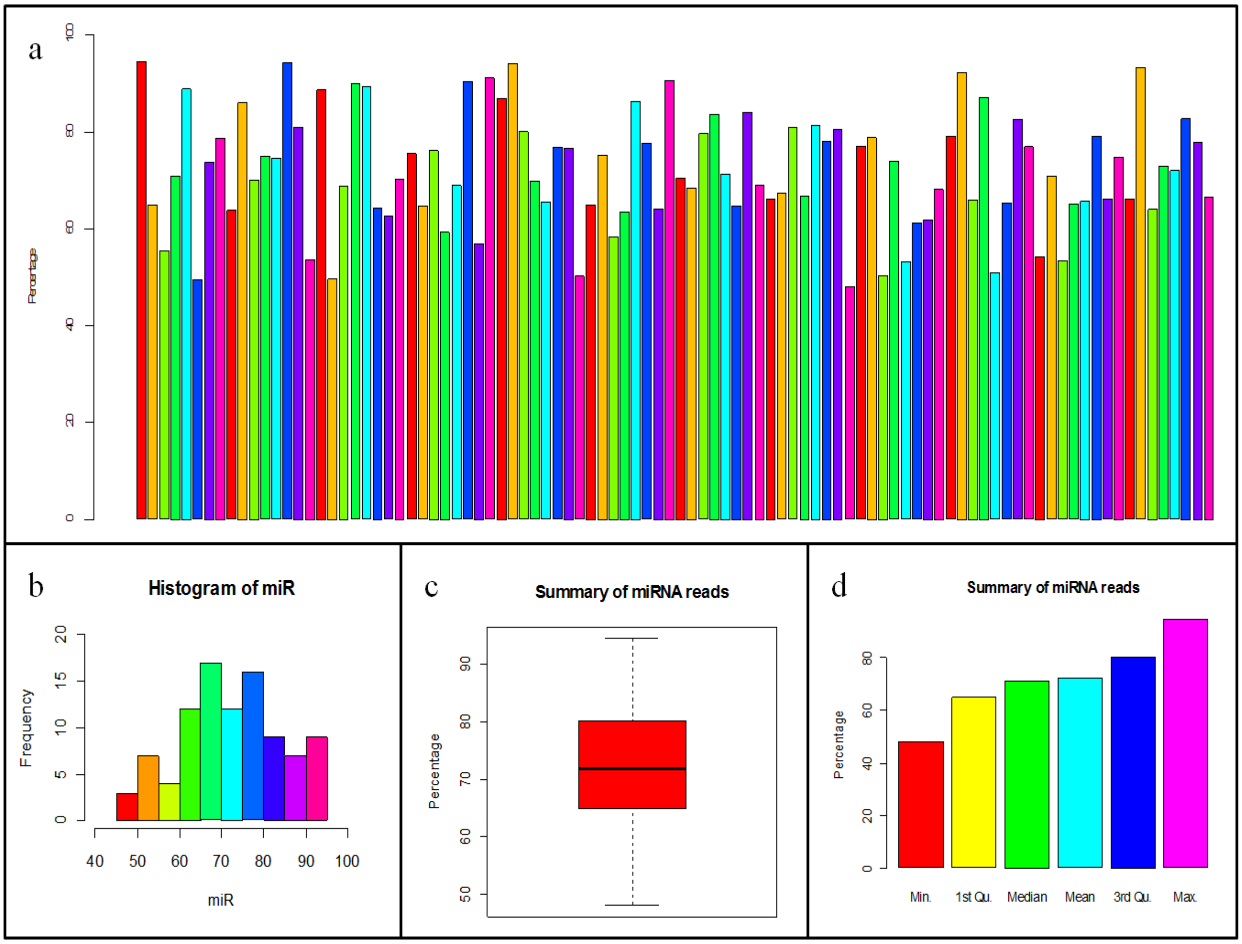

Figure S2. Total miRNA reads in all smRNA summary plots

a. Percentage of miRNA reads in all smRNA reads count was plotted in each sample; b. Histogram graphs showing the distribution of percentage of miRNA count in all samples; c. Box plot of percentage of miRNA reads count in all samples; d. Box plot of the distribution of percentage of miRNA reads count in all samples using the five-number summary method

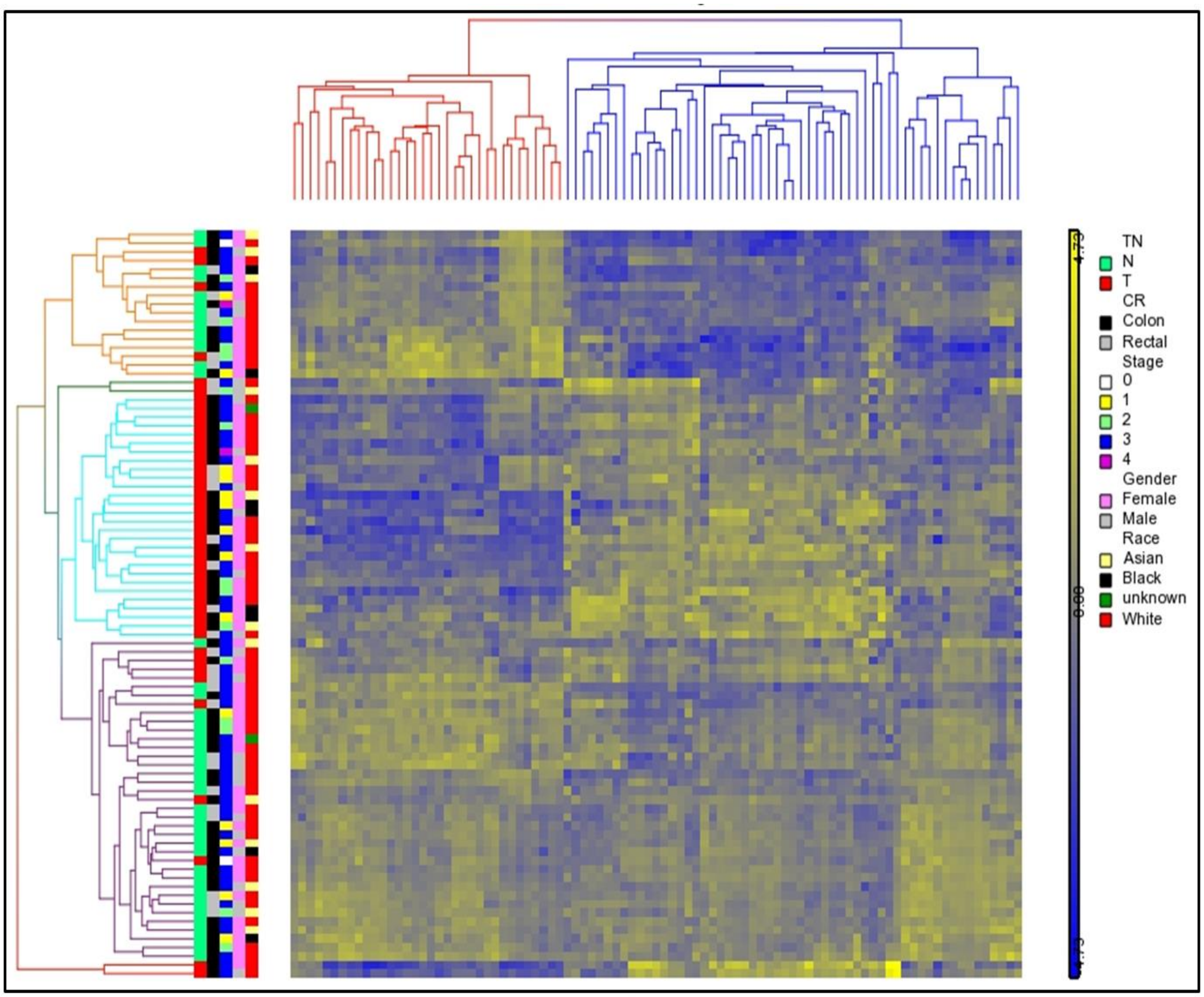

Figure S3. Unsupervised hierarchical cluster analysis using reads count of all miRNAs to classify tumor versus normal tissue

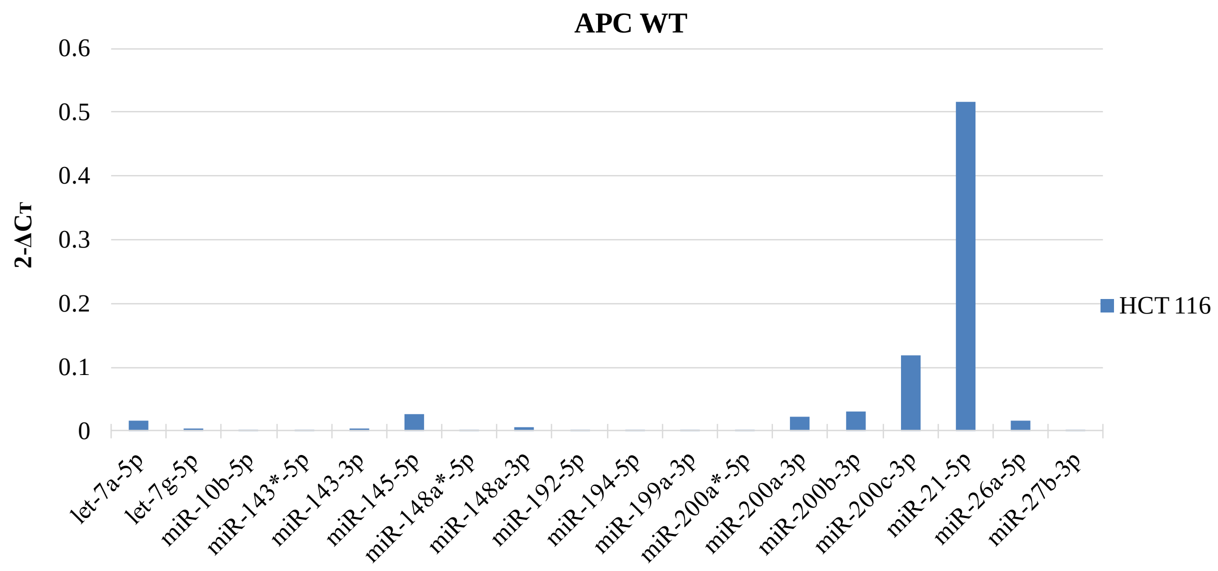

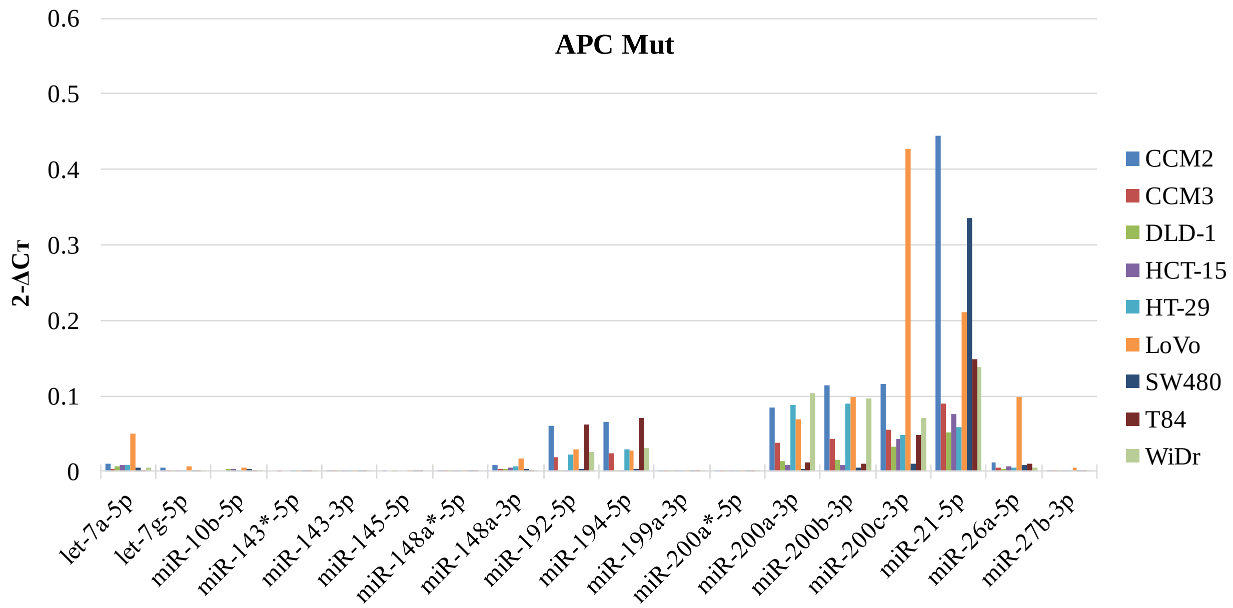

Figure S4. Expression levels of target miRNAs in CRC cell lines with different *APC* gene status detected by Taqman miRNA qPCR.

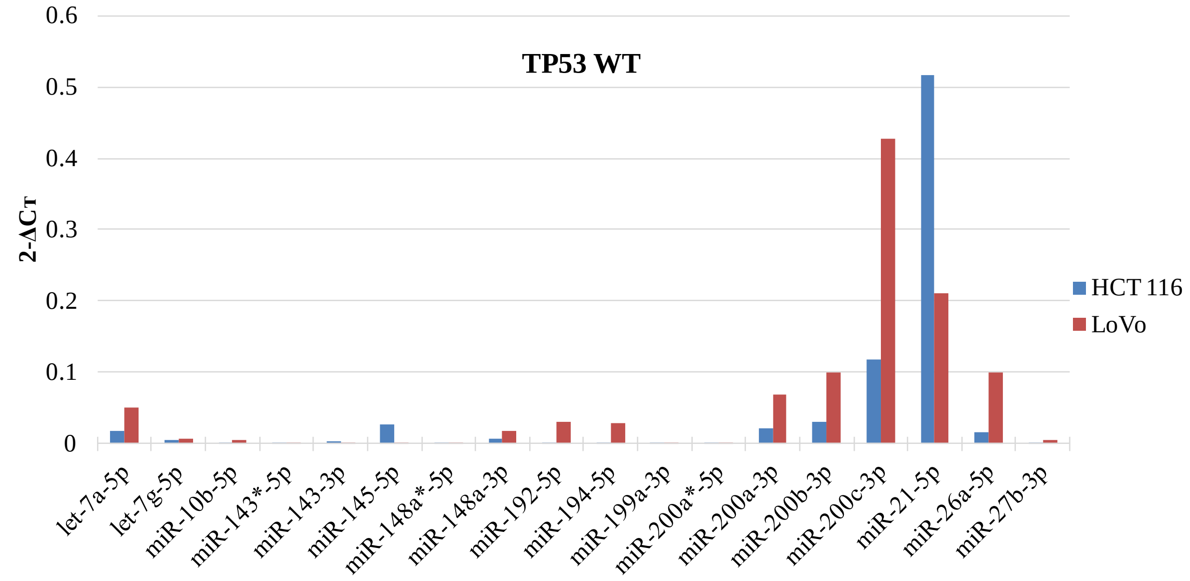

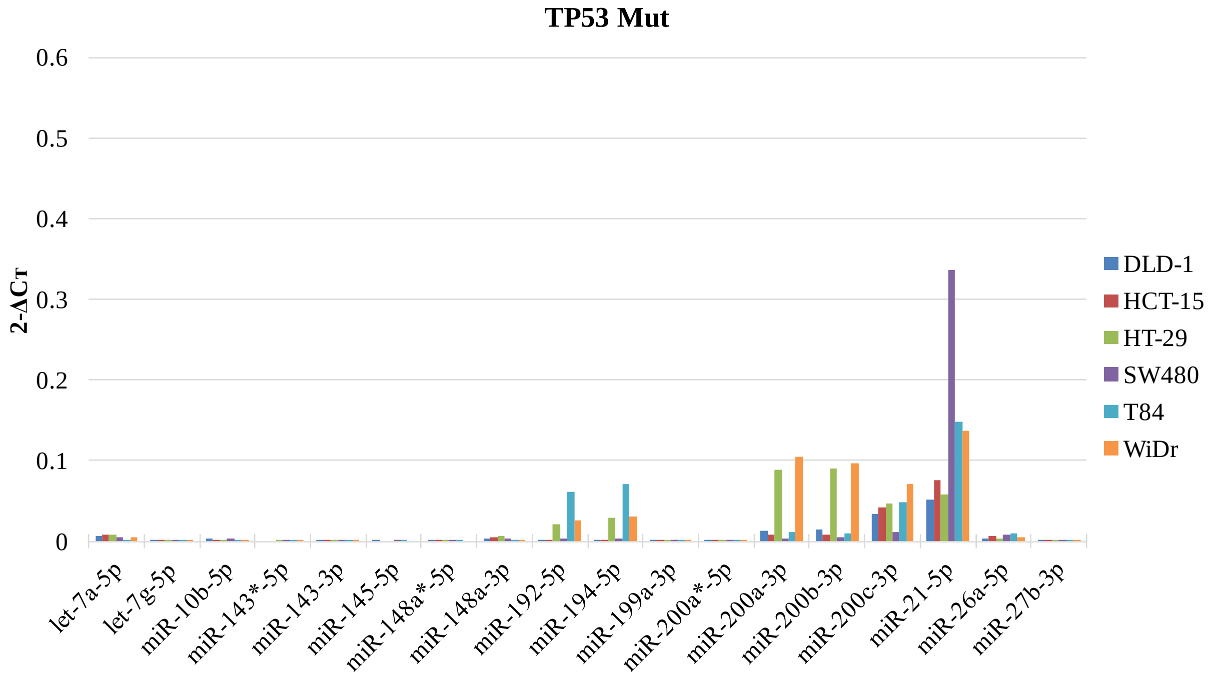

Figure S5. Expression levels of target miRNAs in CRC cell lines with different *p53* gene status detected by Taqman miRNA qPCR.

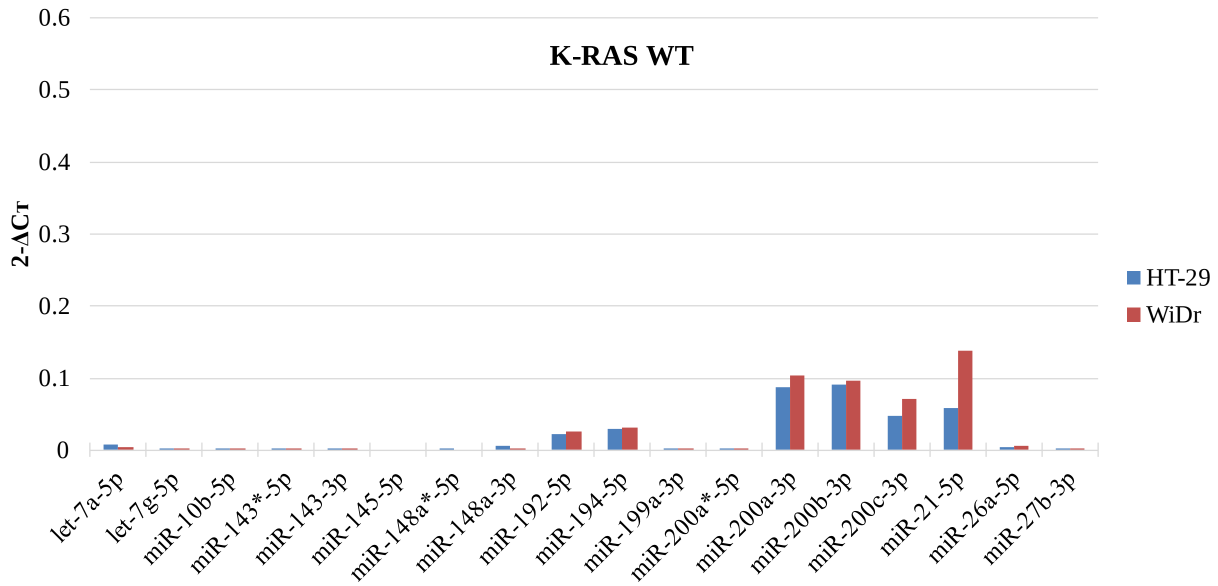

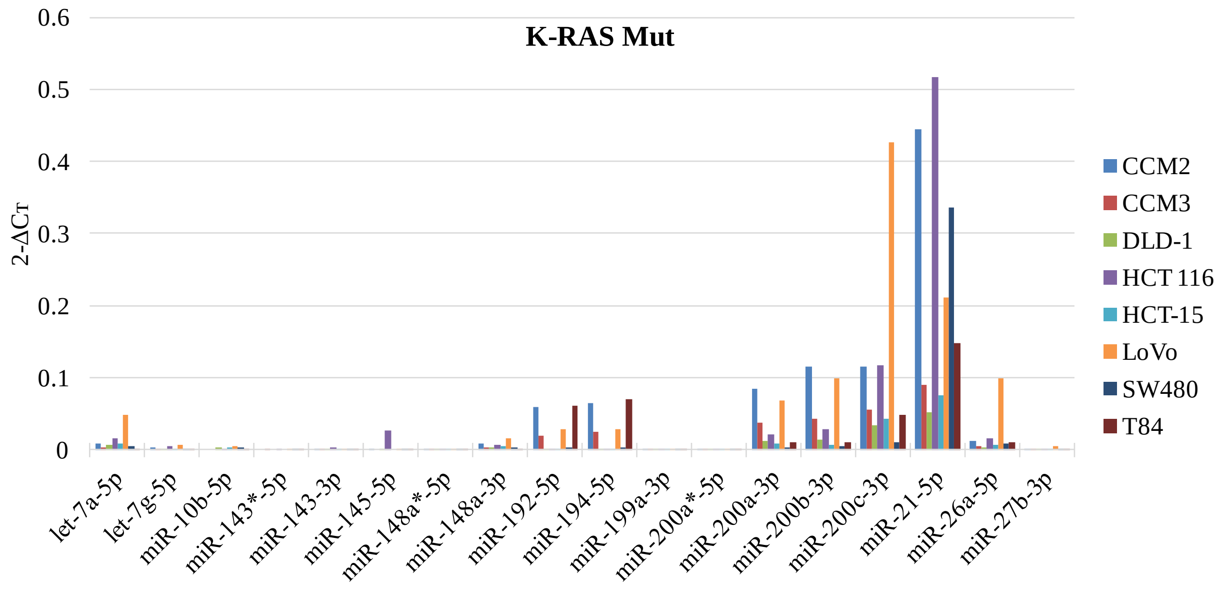

Figure S6. Expression levels of target miRNAs in CRC cell lines with different *k-ras* gene status detected by Taqman miRNA qPCR.
